# Regulation of heart regeneration by LSD1 through suppressing CEND1

**DOI:** 10.7150/thno.110297

**Published:** 2025-05-25

**Authors:** Huahua Liu, Jinling Dong, Shuang Liu, Yuru Luo, Yuan Fang, Hongyu Su, Weihao Xue, Rui Zhou, Wenjun Huang, Baochang Lai, Ying Xiong, Shuangshuang Wang, Lingli Liang, Zhen Wang, Donghong Zhang, Lianpin Wu, Yanmin Zhang, Bin Zhou, John Y-J Shyy, Zuyi Yuan, Yidong Wang

**Affiliations:** 1 Department of Cardiology, First Affiliated Hospital; Cardiometabolic Innovation Center of Ministry of Education, Xi'an Jiaotong University, Xi'an, China; 2 The Institute of Cardiovascular Sciences, School of Basic Medical Sciences; Key Laboratory of Environment and Genes Related to Diseases of Ministry of Education, Xi'an Jiaotong University, Xi'an, China; 3 College of Animal Sciences, Zhejiang University, Hangzhou, Zhejiang, China; 4 Department of Cardiology, The Second Affiliated Hospital, Wenzhou Medical University, Wenzhou, China; 5 Key Laboratory of Precision Medicine to Pediatric Diseases of Shaanxi Province, Shaanxi Institute for Pediatric Diseases, Xi'an Children's Hospital, Affiliated Children's Hospital, Xi'an Jiaotong University, Xi'an, China; 6 Department of Cardiology, the First People's Hospital of Wenling, the Affiliated Hospital of Wenzhou Medical University, Wenling, Zhejiang, China; 7 Department of Physiology and Pathophysiology, School of Basic Medical Sciences, Xi'an Jiaotong University, Xi'an, China; 8 Department of Pediatrics, The University of Chicago, Chicago, IL, USA; 9 Department of Genetics, Albert Einstein College of Medicine, New York, NY, USA; 10 Division of Cardiology, Department of Medicine, University of California, San Diego, La Jolla, CA, USA

**Keywords:** heart regeneration, Cend1, LSD1

## Abstract

**Rationale:** Improving heart regeneration through reactivating cardiomyocyte proliferation holds a great potential for repairing diseased hearts. We recently reported that LSD1-dependent epigenetic repression of Cend1 transcription is prerequisite for cardiomyocyte proliferation and mouse heart development. This study interrogates the potential role of this LSD1-CEND1 axis in heart regeneration and repair.

**Methods:** The cardiomyocyte-specific Lsd1 knockout or overexpression mice, Cend1 null mice and cardiomyocyte-specific Cend1 overexpression mice were used to determine the role of LSD1-CEND1 axis in heart regeneration after experimental injuries. Neonatal and adult mice were subjected to apical resection or left anterior descending coronary artery ligation, respectively, to establish cardiac injury models. Echocardiography and Masson staining were employed to assess cardiac function and histopathology, respectively. The molecular changes were determined using RNA sequencing, quantitative RT-PCR, Western blotting and immunostaining.

**Results:** Cardiomyocyte-specific deletion impeded neonatal heart regeneration, while overexpression of Lsd1 had the opposite effect. RNA sequencing revealed that Cend1, a crucial suppressor of cardiomyocyte cycling, was the most significantly elevated gene induced by Lsd1 loss during heart regeneration. Cardiomyocyte-specific Cend1 overexpression hindered neonatal heart regeneration, while Cend1 loss in nullizygous mice had the opposite effect. Cend1 deletion resulted in gene expression alterations associated with enhanced cardiomyocyte proliferation, neovascularization, and macrophage activation. Furthermore, the cardiac regeneration defect caused by Lsd1 loss was not observed when experiments were performed with mice that were nullizyogus for Cend1. Moreover, we found that either Lsd1 overexpression or Cend1 deletion could promote heart regeneration and repair, and improve cardiac function following experimental myocardial infraction in adult mice.

**Conclusion:** Our results demonstrate that LSD1-dependent suppression of CEND1 is crucial for heart regeneration in neonatal and adult mice after experimental injury. These findings suggest LSD1 activation and CEND1 inhibition as promising therapeutic strategies to enhance endogenous cardiac repair in humans.

## Introduction

In mammals, cardiomyocyte proliferation underpins myocardial tissue growth during embryonic development, and this proliferative activity in cardiomyocytes persists postnatally but is transient and quickly lost 1. In preadolescent humans, it is observed that the majority of cardiomyocytes exit the cell cycle, with only very few cells maintaining the capacity to proliferate. As such, evidence for cardiomyocyte regeneration through cell proliferation and replacement in healthy and diseased hearts remains elusive 2. Notably, clinical studies have shown that the hearts of newborn children with severe myocardial infarction appear to undergo spontaneous repair through a mechanism that is ostensibly associated with myocardial regeneration 3, 4, yet this capacity is lost in adult heart tissues. Moreover, studies of neonatal mouse hearts subjected to apical resection (AR) show extensive tissue repair within three weeks, in part, through proliferation of preexisting cardiomyocytes to replace lost cells 5. Consistent with findings in humans, this capacity for heart regeneration in neonatal mice is lost as soon as 7 days after birth, with what is documented as near-complete loss of cardiac regenerative capacity in adult mice 5. In contrast, studies of lower vertebrates such as zebrafish have shown that adult cardiomyocytes can re-enter the cell cycle, proliferate and repair damaged hearts 6. Such observations have inspired efforts to boost cardiomyocyte proliferation as a mean to promote tissue regeneration in adult mammalian hearts. Indeed, a growing number of studies over the past two decades have provided evidence that the proliferative capacity of adult cardiomyocytes may be reactivated through manipulating key factors that control cardiomyocyte cycling 7-10. Given that heart diseases are a leading cause of death worldwide 11, identifying the key cellular and molecular regulators that control cardiomyocyte cycling and neonatal heart regeneration has clinical implications for developing treatments that leverage such self-repair mechanisms within the adult heart.

One molecular regulator of interest is lysine-specific demethylase 1 (LSD1, also known as KDM1A), the first identified member of a family of demethylases that primarily target mono- and di-methylated lysines on histone 3 (H3K4me1/2 and H3K9me1/2), so as to regulate gene transcription in a positive and negative fashion, respectively 12, 13. Indeed, LSD1 upregulation is well documented in multiple types of cancers and to promote tumor cell proliferation 14-18. By using genetic mouse models, we recently showed that LSD1 is essential for cardiomyocyte proliferation during fetal and neonatal heart development in mice, and that its downstream target gene CEND1 (cell cycle exit and neuronal differentiation 1) controls cardiomyocyte proliferation and myocardial growth. Mechanistically, LSD1 represses *Cend1* transcription by erasing H3K4me2 marks at its promoter 19. Moreover, consistent with its function as an inducer of neuroprogenitor cell cycle exit 20, CEND1 negatively regulates cardiomyocyte cycling through sustaining p53 signaling 19. As a corollary, *Fei et al*. reported that knockdown of *Lsd1* inhibited heart regeneration in neonatal mice 21. Despite these findings, the relevance for a 'LSD1-CEND1 signaling axis' in neonatal and adult cardiac regeneration and the underlying mechanism remains elusive.

In this study, we employed several mouse models to alter expression levels of *Lsd1* or *Cend1* in cardiomyocytes and, through these experiments, we have found that the suppression of CEND1 by LSD1 is crucial for neonatal heart regeneration. We have also conducted experiments to show that either *Lsd1* overexpression or *Cend1* deletion promoted adult heart regeneration and repair, as well as improved cardiac function following myocardial infraction. These results suggest that targeting the LSD1-CEND1 axis could be beneficial as a strategy for triggering heart regeneration in adult mammals.

## Results

### Complementary expression patterns for LSD1 and CEND1 in postnatal murine heart

Initially, we examined the mRNA and protein levels of *Lsd1* and *Cend1* genes in postnatal murine hearts using qPCR and Western blotting (WB), respectively. As shown, the steady-state levels of *Lsd1* mRNA decreased between postnatal days 1 and 7 (P7) and further declined at P14 and then remained unchanged until adulthood (P56) (Figure S1A). Western blotting revealed that steady-state levels of LSD1 were reminiscent of its mRNA expression pattern, with a significant decrease detected between P1 and P7, however, the signal diminished through to adulthood (Figure S1B). In contrast, mRNA levels for *Cend1* in these tissues were significantly elevated at P7 when compared with P1, while expression levels at P28 and P56 were similar to P1 levels (Figure S1C). Western blotting revealed that CEND1 protein levels significantly increased between P1 and P7 and this increase progressed to adulthood (Figure S1D). These findings suggest that the expression patterns of *Lsd1* and *Cend1* are complementary in the postnatal murine heart as it ages.

### *Lsd1* deletion impedes neonatal heart regeneration

**Figure 1:**
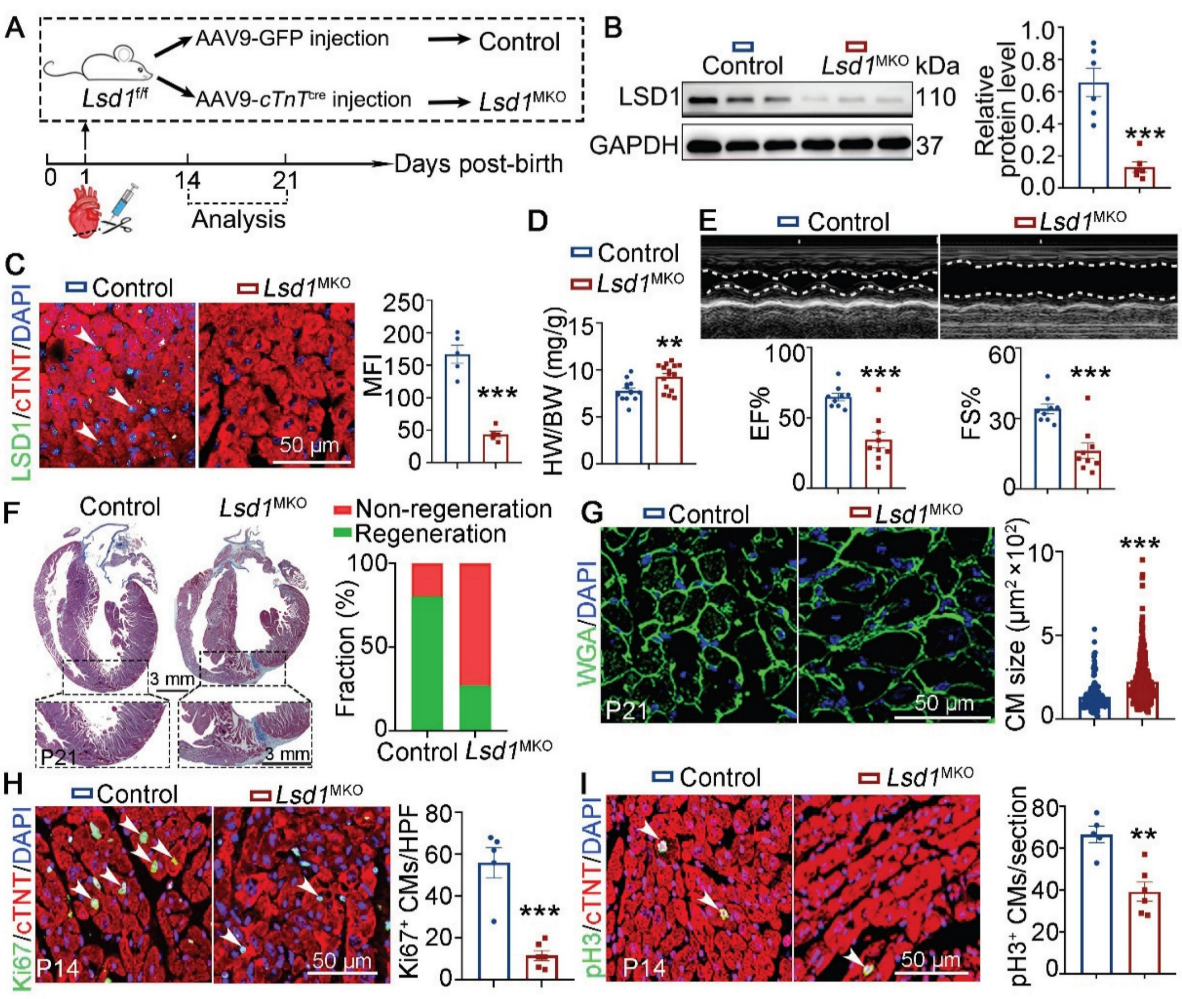
***Lsd1* deletion influences neonatal heart regeneration following AR surgery.** (**A**) Schematic diagram showing experimental procedures. Mice subjected to cardiomyocyte-specific *Lsd1* knockout (*Lsd1*^MKO^) were established by injecting AAV9-*cTNT*^cre^ virus into left ventricles of their hearts at postnatal day 1 (P1). Control mice received AAV-GFP virus. Both *Lsd1*^MKO^ and control mice were subjected to apical resection (AR). (**B**) Western blotting analysis of the LSD1 protein levels in the hearts of P14 mice (n = 6/group). (**C**) Co-immunostaining of LSD1 and cTNT antibodies in P14 hearts (n = 5/group). The mean fluorescence intensity (MFI) was quantified and shown. (**D**) Heart-to-body weight ratios for control (n = 13) and *Lsd1*^MKO^ (n = 15) mice at P21. (**E**) Representative images of echo analysis showing cardiac function of mice at P21. Bar charts showing the values of ejection fraction (EF), fractional shortening (FS). n = 9/group. (**F**) Representative images of Masson staining showing the fibrotic scar in the hearts of control and *Lsd1*^MKO^ mice at P21. The hearts with or without fibrotic scar within apex region were considered non-regeneration or regeneration respectively. Bar chart showing the quantification for the proportion of regeneration and non-regeneration hearts among Control (n = 13) or *Lsd1*^MKO^ (n = 9) group. (**G**) Immunostaining of WGA indicating cardiomyocyte size of control (194 cells from 8 mice) and *Lsd1*^MKO^ (285 cells from 8 mice) mice. (**H**, **I**) Immunostaining with Ki67 (**H**) and pH3 (**I**) antibodies indicating proliferating cells (arrowhead). Bar charts showing quantification for the numbers of Ki67 or pH3 positive cardiomyocytes (CMs) marked by cTNT staining in control (n = 5) and *Lsd1*^MKO^ (n = 6) mice. HPF, high power field. *p < 0.05, **p < 0.01, ***p < 0.001 by unpaired student's *t*-test.

To explore the role of LSD1 in neonatal heart regeneration *in vivo*, we transduced cardiomyocyte-specific loss of *Lsd1* in mice (*Lsd1*^MKO^) through targeted injection of AAV9-*cTnT*^cre^ virus into the left ventricles of conditional *Lsd1*^f/f^ loxp mice at P1, concurrent with AR surgery (Figure 1A). As shown, when analyzed the mice at P14, we found that the LSD1 protein levels in the hearts of *Lsd1*^MKO^ mice were significantly reduced, compared to mice that received control virus (Figure 1B-C). The ratios of heart-to-body weight of P21 *Lsd1*^MKO^ mice were significantly higher than that in age-matched control mice (Figure 1D). Echocardiography analysis revealed that *Lsd1*^MKO^ mice had worse cardiac function, evidenced by reduced ejection fraction (EF) and fraction shortening (FS), compared to control mice (Figure 1E). Masson staining showed that more than 80% of control mice did not show evidence of fibrosis at the apical region of their hearts, while a far lower percentage of *Lsd1*^MKO^ mice had this feature (Figure 1F), suggesting that conditional deletion of *Lsd1* impeded cardiac regeneration. In addition, WGA staining revealed that *Lsd1*^MKO^ mice featured cardiomyocytes that were larger in size than control mice (Figure 1G). Moreover, *Lsd1* deletion significantly inhibited cardiomyocyte proliferation, shown by reduced numbers of Ki67 (a marker for DNA synthesis) and pH3 (a marker for mitosis) positive cardiomyocytes in *Lsd1*^MKO^ mice, compared to control (Figure 1H-I). Therefore, these findings demonstrate that LSD1 is important for neonatal cardiac regeneration following AR surgery.

### *Lsd1* overexpression ameliorates neonatal heart regeneration

**Figure 2:**
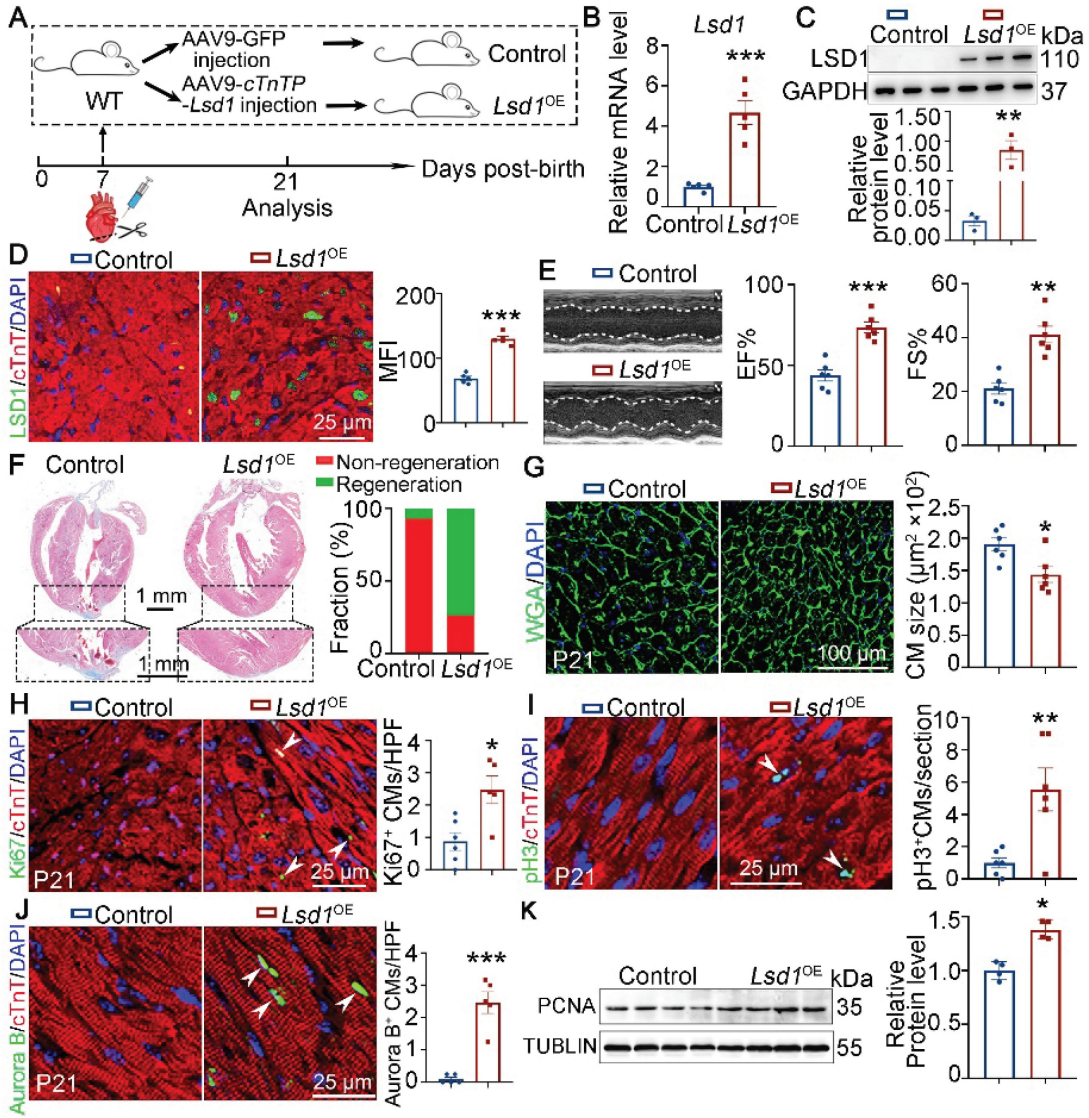
***Lsd1* overexpression ameliorates neonatal heart regeneration following AR surgery.** (**A**) Schematic diagram showing experimental procedures. Mice subjected to cardiomyocyte-specific *Lsd1* overexpression (*Lsd1*^OE^) were generated by injecting AAV9-*cTNTP*-*Lsd1* virus into left ventricle of the hearts of wide type (WT) mice at postnatal day 7 (P7). Control mice received control virus. Both *Lsd1*^OE^ and control mice were subjected to apical resection (AR) at P7. (**B** and **C**) The mRNA and protein levels of LSD1 in the hearts of P21 mice were measured by qPCR (**B**, n = 5) and Western blotting (**C**, n = 3) respectively. (**D**) Co-immunostaining of LSD1 and cTNT antibodies in P21 hearts (n = 5/group). The mean fluorescence intensity (MFI) was quantified and shown. (**E**) Representative images of echo analysis showing cardiac function of mice at P21. Bar charts showing the values of ejection fraction (EF), fractional shortening (FS). n = 6/group. (**F**) Representative images of Masson staining showing the fibrotic scar in the hearts of P21 mice. The hearts with or without fibrotic scar within apex region were considered non-regeneration or regeneration respectively. Bar charts showing quantification for the proportion of regeneration and non-regeneration hearts among control (n = 14) or *Lsd1*^OE^ (n = 15) group. (**G**) Immunostaining of WGA to measure cardiomyocyte sizes in heart tissues from control (826 cells from 6 mice) and *Lsd1*^OE^ (663 cells from 6 mice) mice. (**H**-**J**) Immunostaining with Ki67 (**H**), pH3 (**I**) and Aurora B (**J**) antibodies indicating proliferating cells (arrowheads). Bar charts showing the quantification for the numbers of Ki67, pH3 or Aurora B positive cardiomyocytes (CMs) marked by cTNT staining in control (n = 5-6) and *Lsd1*^OE^ (n = 5-6) mice. (**K**) Western blotting analysis of the PCNA protein levels in the hearts of P21 mice (n = 4/group). HPF, high power field. *p < 0.05, **p < 0.01, ***p < 0.001 by unpaired student's *t*-test.

It is recognized that the capacity for cardiac regeneration in mice is negligible after P7. To investigate whether *Lsd1* gain-of-function might influence the regenerative capacity of neonatal hearts beyond P7, we transduced cardiomyocyte-specific *Lsd1* overexpression (*Lsd1*^OE^) in mouse hearts through injection of AAV9-*Lsd1* virus into the left ventricles of wild type (WT) mice at P7, followed by AR surgery (Figure 2A). The AAV9-*Lsd1* virus is designed to confer over-expression of *Lsd1* under the control of a cardiac-specific *cTnT* promoter. qPCR, Western blotting and immunostaining confirmed successful *Lsd1* overexpression in cardiac tissues, compared to control treatment (Figure 2B-D). When analyzing the treated mice at P21, we found that control mice showed poor cardiac tissue repair at P21, as characterized by insufficient cardiac function and apparent fibrotic scar at the apex region (Figure 2E-F). In contrast, more than 80% of *Lsd1*^OE^ mice featured extensive repair of heart tissue at P21 after AR surgery at P7, and with normal cardiac function and heart histology (Figure 2E-F). Moreover, the size of cardiomyocytes in *Lsd1*^OE^ mice were smaller than that in controls (Figure 2G). Also, Ki67, pH3 and Aurora B (a marker for cytokinesis) immunostaining showed that cardiomyocyte proliferation was significantly increased in *Lsd1*^OE^ mice compared to control (Figure 2H-J). In addition, Western blotting analysis revealed a significant increase in PCNA protein expression (a proliferation marker) in* Lsd1*^OE^ mouse hearts relative to controls (Figure 2K). In contrast, the cell cycle regulator Cyclin D1 protein levels remained unchanged following *Lsd1* overexpression (Figure S2A). Notably, histological and echocardiographic analyses demonstrated that sole overexpression of *Lsd1* had no significant impact on either cardiac growth or function in juvenile mice (Figure S3A-C). Thus, adenovirus-mediated *Lsd1* gain-of-function in P7 hearts prior to AR surgery can induce cardiomyocyte proliferation and promote heart regeneration.

### *Cend1* is repressed by *Lsd1* and inhibits neonatal heart regeneration

**Figure 3:**
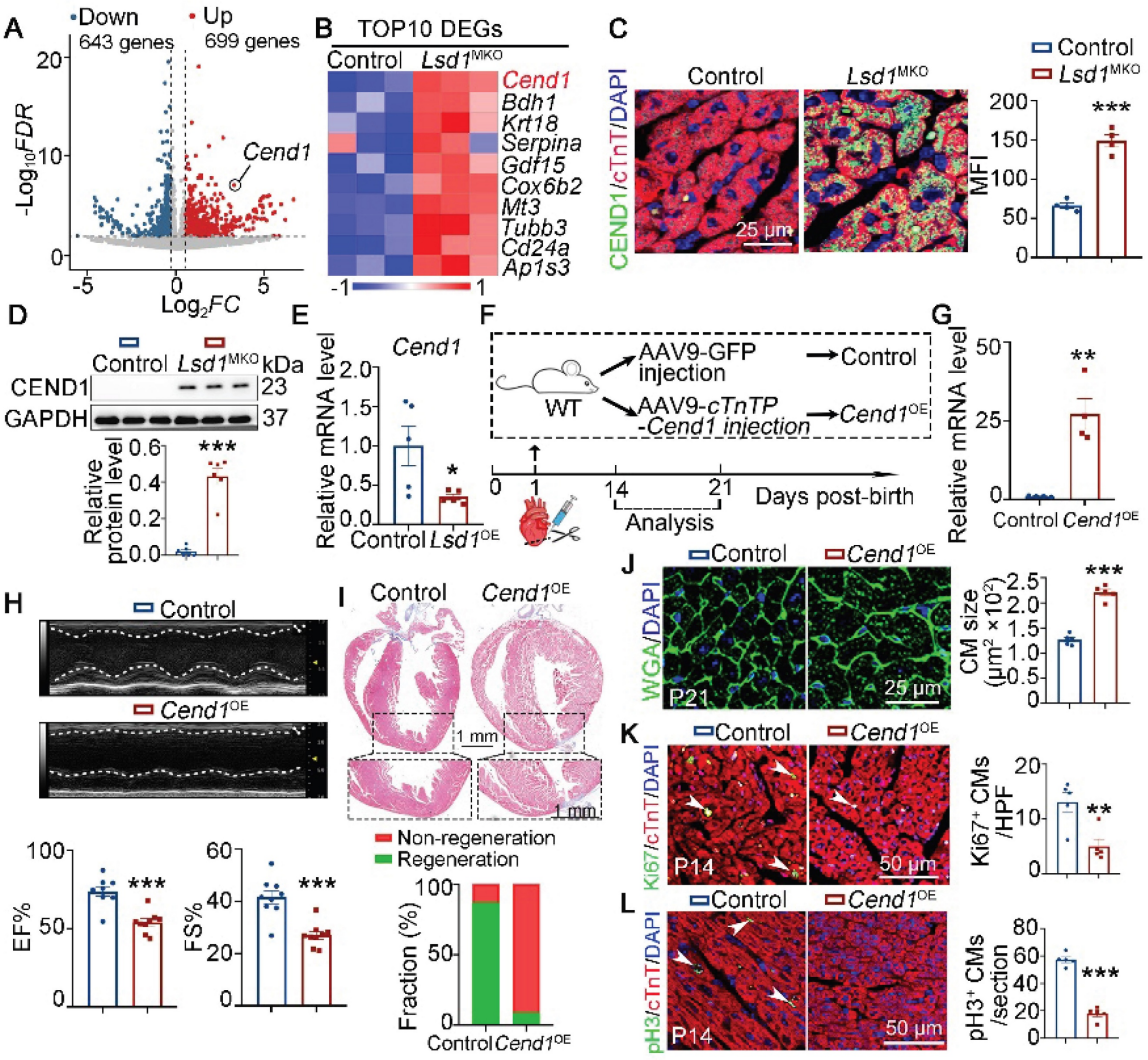
***Cend1* is repressed by *Lsd1* and its overexpression impedes neonatal heart regeneration.** (**A**-**B**) Heart ventricle tissues were isolated from P14 control and *Lsd1*^MKO^ hearts which received apical resection (AR) surgeries at P1. Volcano plots of RNA-sequencing datasets showing differentially expressed genes between two groups (**A**). A Heatmap showing the top 10 upregulated genes induced by *Lsd1* loss (**B**). (**C**) Co-immunostaining of LSD1 and cTNT antibodies in P21 hearts (n = 4/group). The mean fluorescence intensity (MFI) was quantified and shown. (**D**) Western blotting analysis of the CEND1 protein level in P21 hearts (n = 6/group). (**E**) qPCR analysis of the *Cend1* mRNA level in P21 hearts (n = 5/group). (**F**) Schematic diagram showing experimental procedures. Mice subjected to cardiomyocyte-specific *Cend1* overexpression (*Cend1*^OE^) were generated by injecting AAV9-*cTNTP*-*Cend1* virus into the left ventricles of the hearts of wide type (WT) mice at P1, while the mice received AAV9-GFP virus were used as controls. Both *Cend1*^OE^ and control mice were subjected to AR surgeries at P1. (**G**) qPCR analysis of the *Cend1* mRNA level in the hearts of P14 mice (n = 4/group). (**H**) Representative images of echo analysis showing cardiac function of mice at P21. Bar charts showing the values for ejection fraction (EF) and fractional shortening (FS). n = 9/group. (**I**) Representative images of Masson staining showing the fibrotic scar in the hearts of P21 mice. The hearts with or without fibrotic scar within apex region were considered non-regeneration or regeneration respectively. Bar chart showing the quantification for the proportion of regeneration and non-regeneration hearts among control (n = 9) or *Cend1*^OE^ (n = 11) group. (**J**) WGA staining indicating cardiomyocyte size of control (878 cells from 5 mice) and *Cend1*^OE^ (722 cells from 5 mice) mice. (**K**, **L**) Immunostaining with Ki67 (**K**) and pH3 (**L**) antibodies indicating proliferating cells (arrowheads). Bar charts showing the quantification for the numbers of Ki67 or pH3 positive cardiomyocytes (CMs) marked by cTNT staining in control (n = 5) and *Cend1*^OE^ (n = 5) mice. HPF, high power field. *p < 0.05, **p < 0.01, ***p < 0.001 by unpaired student's *t*-test.

To explore the downstream targets of LSD1 that increase heart regeneration, we analyzed the transcriptomic profiles of heart tissues collected from P14 control and *Lsd1*^MKO^ mice that received AR surgery at P1. As shown, the targeted *Lsd1* loss in the heart tissues of *Lsd1*^MKO^ mice led to 1342 differentially expression genes. Among these, we identified 699 and 643 genes that were upregulated and downregulated, respectively (Figure 3A). We recently identified that *Cend1* is a key negative regulator of cardiomyocyte cycling suppressed by *Lsd1* during heart development 19 and, interestingly, the RNA-seq results showed that *Cend1* was the most significantly elevated gene resulting from *Lsd1* loss concurrent with AR surgery in our experiment (Figure 3B). This upregulation was confirmed at the translational level by immunostaining and Western blotting (Figure 3C-D). In a parallel experiment in which *Lsd1* was conditionally overexpressed in the setting of AR surgery (that is, in *Lsd1*^OE^ mice), *Cend1* was downregulated in the hearts of these mice, compared to controls (Figure 3E, Figure S4A). Given that *Cend1* inhibits cardiomyocyte proliferation during heart development 19, our current results suggest that *Cend1* may function as a crucial negative regulator of cardiomyocyte proliferation and heart regeneration. To test this hypothesis, we induced cardiomyocyte-specific *Cend1* overexpression (*Cend1*^OE^) mice through injection of AAV9-*Cend1* virus into the left ventricles of WT mice at P1, followed by AR surgery (Figure 3F). The AAV9-*Cend1* virus confers over-expression of *Lsd1* under the control of a cardiac-specific *cTnT* promoter. qPCR and immunostaining confirmed significant *Cend1* overexpression in cardiomyocytes (Figure 3G, Figure S4B). In this experiment, mouse heart repair at P21 was poor for *Cend1*^OE^ mice compared to control, as characterized by poor cardiac function and fibrotic scar at the apex region, in comparison to comprehensive heart repair in over 90% of control mice (Figure 3H-I). Moreover, the cardiomyocyte size in *Cend1*^OE^ mice was significantly larger, compared to that in controls (Figure 3J). Furthermore, immunostaining revealed that *Cend1* overexpression resulted in significant reductions in Ki67 and pH3, indicative decrease in cardiomyocyte proliferation (Figure 3K-L). Notably, histological and echocardiographic analyses revealed that* Cend1* overexpression had no significant impact on either cardiac growth or function in juvenile mice under physiological conditions (Figure S4C-E).

**Figure 4:**
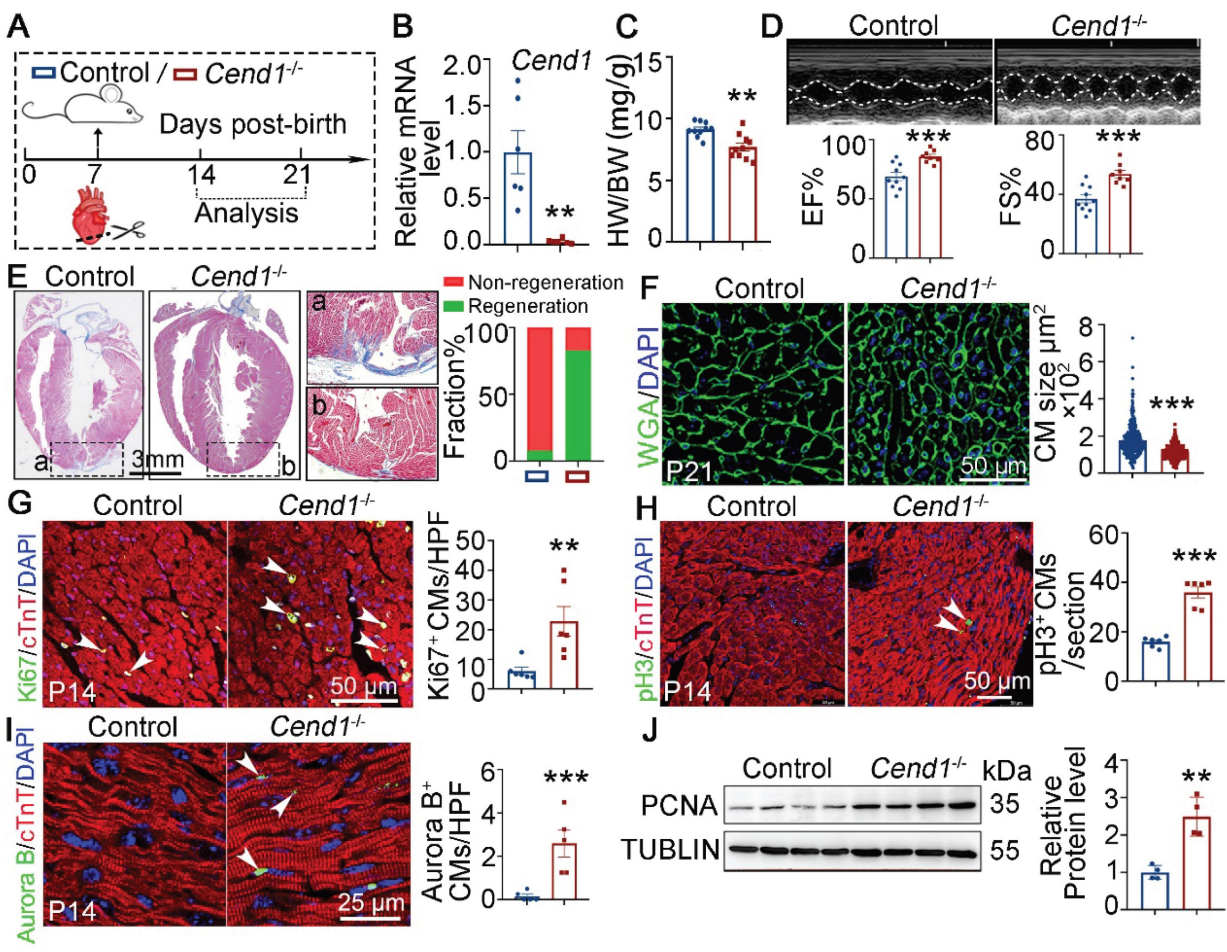
** Loss of *Cend1* is permissive for juvenile heart regeneration.** (**A**) Schematic diagram showing experimental procedures. Control (WT) and *Cend1*^-/-^ mice were subjected to apical resection (AR) surgeries at P7. (**B**) qPCR analysis of the *Cend1* mRNA level in P14 hearts (n = 6/group). (**C**) Heart-to-body weight ratios of mice at P21 (n = 10/group). (**D**) Representative images of echo analysis showing cardiac function of mice at P21. Bar charts showing the values of ejection fraction (EF) and fractional shortening (FS) for control (n = 10) and *Cend1*^-/-^ (n = 8) mice. (**E**) Representative images of Masson staining showing the fibrotic scar in the hearts of P21 mice. The hearts with or without fibrotic scar within apex region were considered non-regeneration or regeneration respectively. Bar charts showing the quantification for the proportion of regeneration and non-regeneration hearts among each group (n = 23/group). (**F**) Immunostaining of WGA indicating cardiomyocyte size of control (345 cells from 8 mice) and *Cend1*^-/-^ (360 cells from 8 mice) mice. (**G**-**I**) Immunostaining with Ki67 (G), pH3 (**H**) and Aurora B (**I**) antibodies indicating proliferating cells (arrowheads). Bar charts showing the quantification for the numbers of Ki67, pH3 or Aurora B positive cardiomyocytes (CMs) marked by cTNT staining in control (n = 5-6) and *Cend1*^-/-^ (n = 5-6) mice. (**J**) Western blotting analysis of the PCNA protein levels in P21 hearts (n = 4/group). HPF, high power field. *p < 0.05, **p < 0.01, ***p < 0.001 by unpaired student's *t*-test.

Next, we used nullizygous *Cend1*^-/-^ mice to explore if *Cend1* repression is necessary for the regeneration of neonatal heart. First, we observed that under physiological conditions, *Cend1*^-/-^ mice showed no significant differences in cardiac histology and function compared to control mice (Figure S4F-H). Subsequently, we subjected both *Cend1*^-/-^ and wild type control mice to AR surgery at P7 and assessed cardiac repair at P14 and P21 (Figure 4A). qPCR analysis confirmed that *Cend1* transcripts were undetectable in *Cend1*^-/-^ hearts (Figure 4B). The heart-to-body weight ratios of *Cend1*^-/-^ mice were significantly reduced, compared to controls at P21 (Figure 4C). Furthermore, more than 90% of control mice exhibited poor heart repair at P21, evidenced by impaired cardiac function and obvious fibrotic scar at the apex region (Figure 4D-E). In contrast, over 80% of *Cend1*^-/-^ mice featured significant heart repair, characterized by normal cardiac function and histological features (Figure 4D-E). Moreover, cardiomyocytes in *Cend1*^-/-^ mice were significantly smaller than that those in controls **(**Figure 4F**)**. In agreement, levels of Ki67, pH3 and Aurora B were significantly increased in *Cend1*^-/-^ mice (Figure 4G-I). Furthermore, the PCNA protein levels were markedly increased in the hearts of *Cend1*^-/-^ mice compared to controls (Figure 4J)**.** In contrast, Cyclin D1 protein levels remained unchanged following *Cend1* deletion (Figure S2B). Together, these findings suggest that *Cend1* repression is critical for heart regeneration in neonatal mice.

**Figure 5:**
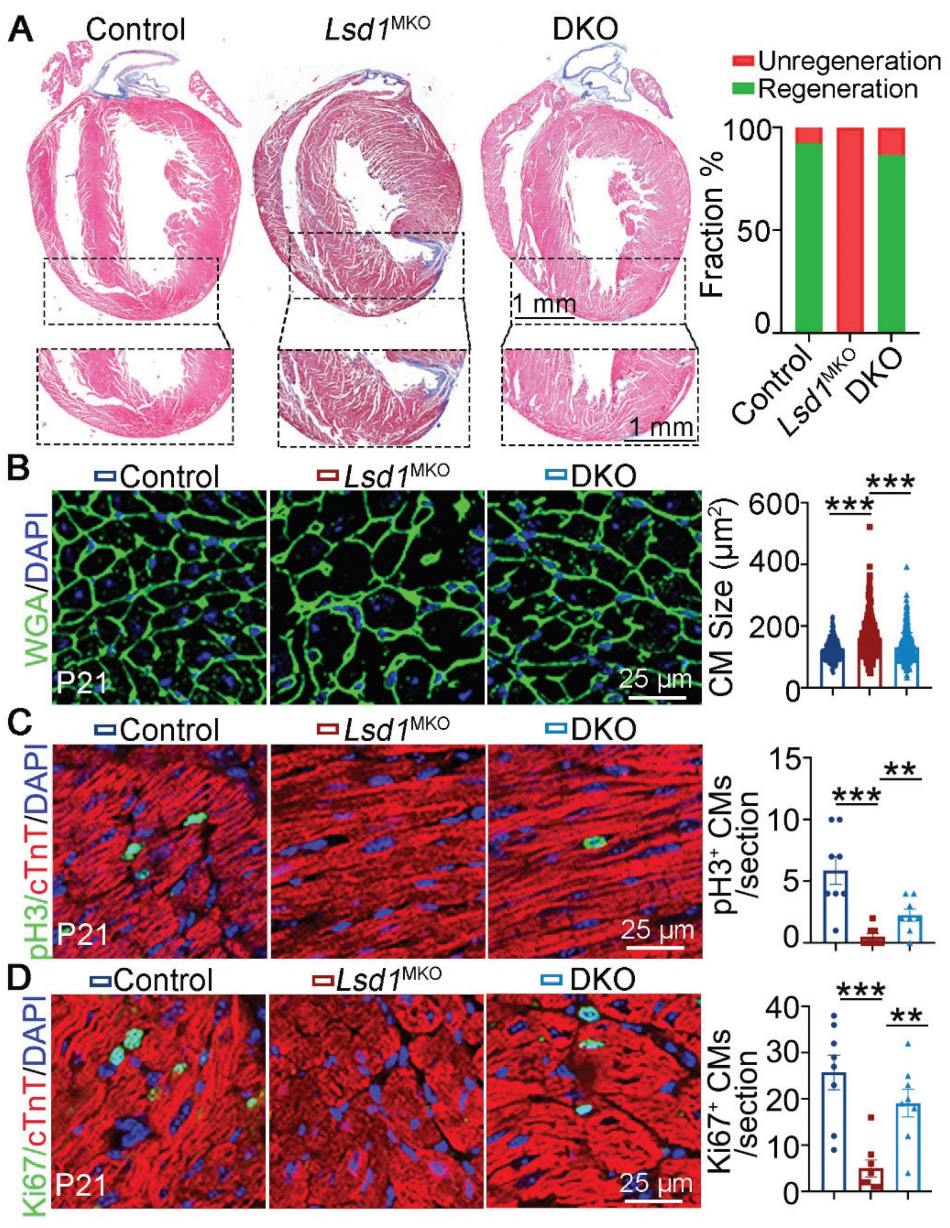
***Cend1* deletion rectifies the regeneration defects caused by *Lsd1* loss.** The *Lsd1*^f/f^;*Cend1*^-/-^ and *Lsd1*^f/f^ mice at postnatal day 1 (P1) were injected with AAV9-*cTNT*^cre^ virus to generate *Lsd1* and* Cend1* double knockout (DKO) and *Lsd1* single knockout mice (*Lsd1*^MKO^) respectively, while *Lsd1*^f/f^ mice received AAV9-GFP virus and these were used as controls. The mice were subjected to apical resection (AR) surgeries at P1 and analyzed at P21. (**A**) Representative images of Masson staining showing the fibrotic scar in the hearts of P21 mice. The hearts with or without fibrotic scar within apex region were considered non-regeneration or regeneration respectively. Bar charts showing the quantification for the proportion of regeneration and non-regeneration hearts among control (n = 13), *Lsd1*^MKO^ (n = 9) or DKO (n = 15) group. (**B**) Immunostaining of WGA indicating cardiomyocyte size of control (330 cells from 8 mice), *Lsd1*^MKO^ (360 cells from 8 mice) and DKO (330 cells from 7 mice) mice. (**C**, **D**) Immunostaining with pH3 (**C**) and Ki67 (**D**) antibodies indicating proliferating cells (arrowhead). Bar charts showing the quantification for the numbers of Ki67 or pH3 positive cardiomyocytes (CMs) marked by cTNT staining (n = 8/group). *p < 0.05, **p < 0.01, ***p < 0.001 by One-way ANOVA with Tukey tests.

To determine the mechanism by which *Cend1* loss promoted neonatal heart regeneration, we performed RNA-sequencing to compare transcriptomic variations between control and *Cend1*^-/-^ heart tissue at P14. Among the 3136 differentially expressed genes, 1932 were upregulated and 1204 were downregulated (Figure S5A). GO analysis revealed that these upregulated genes were involved in cell cycle regulation, angiogenesis and macrophage activation, while the downregulated genes were associated with sarcomere, cardiomyocyte differentiation and cardiac muscle contraction (Figure S5B). Notably, heart tissues ablated with* Cend1* showed elevated expression levels for M2 macrophages markers such as *ll1ra*, *CD163*, *Mrc1*, *Pecam1*, *Ccl24*, *Igf1*, *Arg1*, *CD86*, *Cxcl1* (Figure S5C). In addition, GSEA assays revealed that the DEGs associated with *Cend1* knockout were enriched in vascular endothelial growth factor production, angiogenesis and extracellular matrix (ECM) regulation (Figure S5D). In line with these transcriptomic changes, *Cend1*^-/-^ hearts had more capillary vessels marked by VE-cadherin and reparative macrophages immunostained by CD206 (Figure S5E-F). Notably, the mRNA and protein levels for Agrn, OPN (encoded by *Spp1*) and Versican, three extracellular matrix proteins known to promote heart regeneration 22-24, were significantly upregulated in the heart tissues of *Cend1*^-/-^ mice compared to controls (Figure 5G-I). These results demonstrate that *Cend1* loss leads to transcriptomic changes associated with cardiomyocyte proliferation, angiogenesis and macrophage polarization.

### LSD1 supports neonatal heart regeneration through suppressing CEND1

To determine the functional importance for LSD1-dependent repression of *Cend1 in* neonatal heart regeneration, we generated compound mutant, *Lsd1*^f/f^; *Cend1*^-/-^ mice homozygous for *Cend1* knockout and the conditional (loxp) *Lsd1* allele (hereafter refers to DKO). With these DKO mice, we injected AAV9-*cTnT*^cre^ virus into their left ventricles at P1 when AR surgery was performed simultaneously. Masson staining of tissues showed that the majority of control and DKO mice had extensive heart repair at P21, in contrast to all *Lsd1*^MKO^ mice that failed to do so, as evidenced by the presence of fibrotic scars at the apex regions of hearts (Figure 5A). When measuring the cardiomyocyte size, we found that it was comparable between DKO and control mice, while cardiomyocytes were significantly larger in *Lsd1*^MKO^ mice (Figure 5B). Moreover, the Ki67 and pH3 positive cells were significantly reduced in *Lsd1*^MKO^ mice compared to control. However, these marker expression in DKO mice was significantly elevated compared to *Lsd1*^MKO^ mice, manifesting enhanced proliferation in DKO mice (Figure 5C-D). Thus, conditional loss of LSD1 in *Cend1* knockout mice is beneficial for AR surgery-induced neonatal heart regeneration.

### LSD1 suppression of CEND1 is beneficial for adult heart repair

**Figure 6:**
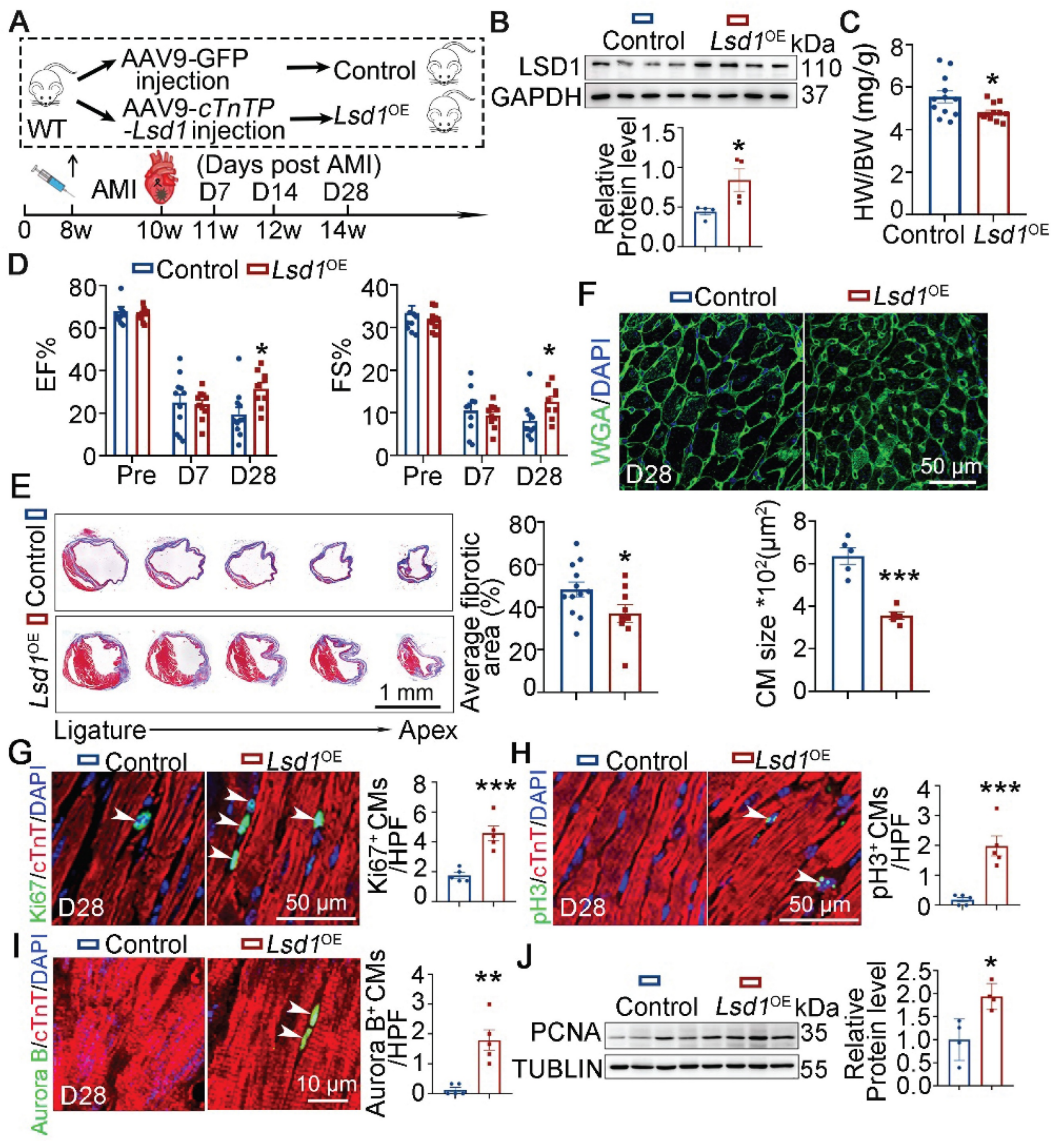
***Lsd1* overexpression promotes adult heart repair.** (**A**) Schematic diagram showing experimental procedures. Adult wild type (WT) mice were injected with AAV9-* cTnTP*-*Lsd1* via tail vein to generate mice with cardiomyocyte-specific *Lsd1* overexpression (*Lsd1*^OE^), while mice that received AAV9-GFP were used as controls. Two weeks after virus injection, the mice were subjected to generation of acute myocardial infraction (AMI) model by left anterior descending artery ligation surgeries. (**B**) The protein levels of LSD1 in the hearts of D7 mice were measured by Western blotting (n = 4/group). (**C**) Heart-to-body weight ratios for control (n = 12) and *Lsd1*^OE^ (n = 12) mice at 4 weeks post AMI surgeries. (**D**) Bar charts showing the values of ejection fraction (EF) and fractional shortening (FS) of control (n = 10-11) and *Lsd1*^OE^ (n = 10-11) mice at indicated times. (**E**) Representative images of Masson staining showing the fibrotic scar in the hearts. The bar chart showing the quantification for the proportion of fibrotic scar among total myocardium in control (n = 11) and* Lsd1*^OE^ (n = 9) mice. (**F**) Immunostaining for WGA indicating cardiomyocyte size of control (481 cells from 5 mice) and* Lsd1*^OE^ (681 cells from 5 mice) mice. (**G**-**I**) Immunostaining with Ki67, pH3 and Aurora B antibodies indicating proliferating cells (arrowheads). Bar charts showing the quantification for the numbers of Ki67 (**G**), pH3 (**H**) or Aurora B (**I**) positive cardiomyocytes (CMs) marked by cTNT staining in control (n = 5) and *Lsd1*^OE^ (n = 5) mice. (**J**) Western blotting analysis of the PCNA protein levels in D28 hearts (n = 4/group). HPF, high power field. *p < 0.05, ***p < 0.001 by unpaired student's *t*-test.

Given that it mediated neonatal heart regeneration, we asked whether the LSD1-CEND1 axis might be relevant to adult heart regeneration and repair. To achieve this, we administered AAV-*Lsd1* virus to adult (8 weeks) WT mice that confers *Lsd1* transgene expression under the control of a *cTnT* promoter, to generate *Lsd1*^OE^ mice. In parallel, control mice received AAV-GFP virus. Next, these virus-injected mice were subjected to acute myocardial infraction (AMI) through left anterior descending coronary artery ligation (LAD) (Figure 6A). The successful overexpression of *Lsd1* in the hearts of *Lsd1*^OE^ mice was confirmed through Western blotting analysis (Figure 6B). The heart-to-body weight ratios of *Lsd1*^OE^ mice were significantly smaller compared to controls (Figure 6C). Also, the cardiac function of *Lsd1*^OE^ mice was significantly improved over control mice 28 days after AMI surgeries, evidenced by increased values of EF and FS (Figure 6D and Figure S6A). Consistently, *Lsd1*^OE^ mice showed reduction in fibrotic scars in their heart tissues when compared to controls (Figure 6E). WGA staining further showed that the cardiomyocyte size in *Lsd1*^OE^ mice was significantly smaller than that in control hearts (Figure 6F). Consistently, *Lsd1* overexpression following AMI induced significant cardiomyocyte proliferation, compared to AMI alone in adult mice (Figure 6G-I). In addition, the PCNA protein levels were significantly increased in the hearts *Lsd1*^OE^ mice compared to controls (Figure 6J)**.** In contrast, Cyclin D1 protein levels were unaffected by *Lsd1* overexpression (Figure S2C). It is important to note that *Lsd1* overexpression had no detectable impact on cardiac structure or function under baseline conditions (Figure S3D-F).

**Figure 7:**
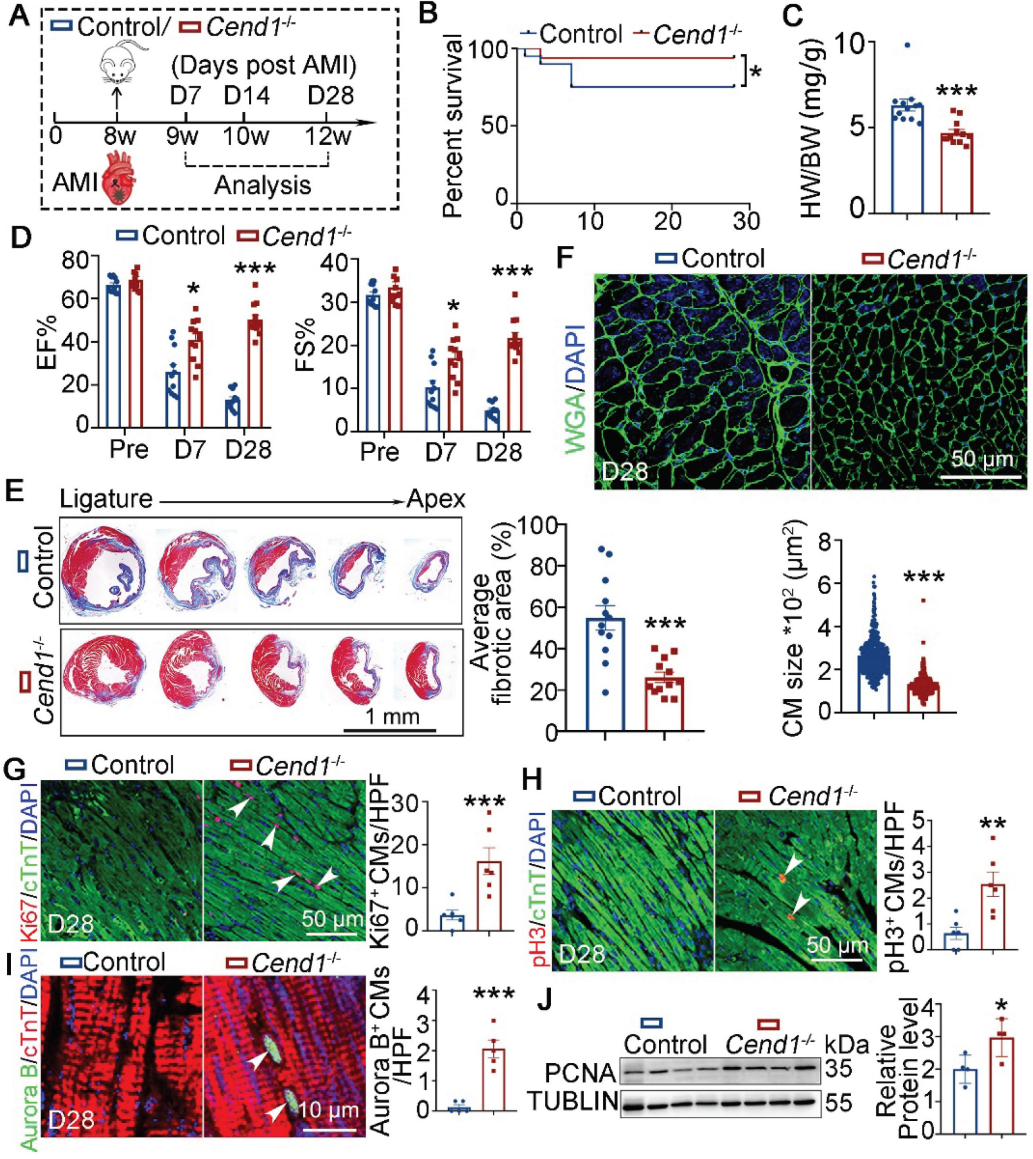
** Adult heart repair following experimental AMI is enhanced in *Cend1* knockout mice.** (**A**) Schematic diagram showing experimental procedures. Adult wild type (WT, control) and *Cend1*^-/-^ mice were subjected to generation of acute myocardial infarction (AMI) model by left anterior descending artery ligation surgeries. (**B**) A chart showing the survival curve of control (n = 20) and *Cend1*^-/-^ (n = 16) mice. (**C**) Heart-to-body weight ratios for mice at 28 days after AMI. n = 12/group. (**D**) Bar charts showing the values of ejection fraction (EF) and fractional shortening (FS) of control (n = 12) and *Cend1*^-/-^ (n = 12) mice at indicated times. (**E**) Representative images of Masson staining showing the fibrotic scar in the hearts. Bar chart showing the quantification for the proportion of fibrotic scar among total myocardium in control (n = 12) and* Cend1*^-/-^ (n = 12) mice. (**F**) Immunostaining of WGA indicating cardiomyocyte size of control (479 cells from 8 mice) and* Cend*^-/-^ (435 cells from 8 mice) mice. (**G**-**I**) Immunostaining with Ki67, pH3 and Aurora B antibodies indicating proliferating cells (arrowheads). Bar charts showing the quantification for the numbers of Ki67 (**G**), pH3 (**H**) or Aurora B (**I**) positive cardiomyocytes (CMs) marked by cTNT staining in control (n = 5-6) and *Cend*^-/-^ (n = 5-6) mice. (**J**) Western blotting analysis of the PCNA protein levels in D28 hearts (n = 4/group). HPF, high power field. *p < 0.05, **p < 0.01, ***p < 0.001 by unpaired student's *t*-test.

For functional relevance, we performed LAD ligation on adult* Cend1*^-/-^ (n = 20) and WT (Control, n = 16) mice (Figure 7A). At 28 days after AMI, the survival rates of WT mice were lower (at 22.5%) compared to *Cend1*^-/-^ mice, where only one *Cend1*^-/-^ mouse died after AMI (Figure 7B). Moreover, the heart-to-body weight ratios of *Cend1*^-/-^ mice were significantly different from those of WT mice (Figure 7C). Moreover, *Cend1*^-/-^ mice had increased EF and FS values (Figure 7D, Figure S6B), indicating that *Cend1* loss led to enhanced recovery of cardiac function following AMI. Consistently, *Cend1*^-/-^ mice had significant less fibrotic scars in their hearts compared to controls (Figure 7E). In addition, the cardiomyocyte size in *Cend1*^-/-^ mice was significantly smaller than it in controls (Figure 7F). Concomitant with the improved heart repair phenotypes in *Cend1*^-/-^ mice after AMI, immunostaining revealed elevated expression of Ki67, pH3 and Aurora B in cardiomyocytes (Figure 7G-I). Like *Lsd1*^OE^ mice, *Cend1*^-/-^ mice exhibited significant upregulation of PCNA protein levels in myocardial tissues compared to controls (Figure 7J). In contrast, Cyclin D1 protein levels were comparable between two groups (Figure S2D). Taken altogether, our findings suggest that LSD1 induces cardiomyocyte proliferation and promotes adult heart repair likely through downregulation of the cell cycle inhibitor *Cend1*.

## Discussion

We recently demonstrated that LSD1-dependent epigenetic suppression of *Cend1* is essential for cardiomyocyte proliferation and heart development in mice 19. Here we extended that study to find that repression of *Cend1* by LSD1 is crucial for cardiomyocyte proliferation during neonatal and adult heart regeneration after experimental injury (Figure S7).

The neonatal mouse heart subjected to either AR or LAD surgeries can extensively repair itself through a mechanism that involves proliferation of existing cardiomyocytes 5, 25, but this repair capacity is largely lost after P7 because the majority of cardiomyocytes exit cell cycle beyond this postnatal stage 5 through mechanism that so far has not been well characterized. In our current study, we found that the expression level of LSD1 was high in the hearts of P1 mice but decreased after P7, while the level of CEND1 featured a complementary pattern of expression. Indeed, their dynamic expression pattern is observed during the critical period for neonatal heart tissue regeneration, and this motivated our investigation that LSD1-CEND1 signaling could be relevant to cardiomyocyte cell cycle exit and proliferation in the context of injury and repair in early and, potentially, adult hearts. Our hypothesis is supported by the evidence in our current study that collectively indicates that, loss of *Lsd1* or overexpression of *Cend1* in heart tissues can impede heart regeneration in neonatal P1 mice following AR surgery, while *Lsd1* overexpression or loss of *Cend1* significantly promote heart regeneration in juvenile P7 mice. This is consistent with previous findings that *Lsd1* knockdown in mice curtails neonatal heart regeneration 21.

In our recent report, we identified that *Cend1* was upregulated following *Lsd1* loss and, furthermore, genetic deletion of *Cend1* could correct the cardiomyocyte proliferation defect and embryonic lethality in cardiomyocyte-specific *Lsd1* knockout mouse embryos 19. In line with our previous findings, RNA sequencing results in the current study identified *Cend1* as the most significantly upregulated gene in the hearts of *Lsd1* knockout mice that were subjected to AR surgeries. Moreover, genetic deletion of *Cend1* was able to rescue the impaired cardiac regeneration in *Lsd1* knockout mice. Mechanistically, our recent work demonstrated that LSD1 represses *Cend1* transcription through H3K4me2 demethylation at its promoter 19. Emerging evidence highlights the context-dependent nature of LSD1 function, wherein its biological outcomes are dictated by stage-specific binding partners. In embryonic stem cells, LSD1-CoREST complexes maintain pluripotency by silencing differentiation genes 26, 27. During myeloid lineage commitment, LSD1-NuRD interactions promote terminal differentiation 28. Our findings extend this paradigm to cardiac regeneration, where LSD1-mediated *Cend1* suppression appears to facilitate proliferative responses. However, the specific co-factors recruiting LSD1 to the *Cend1* locus in neonatal versus adult cardiomyocytes remain undefined.

Notably, CEND1 overexpression not only inhibited proliferation but also induced cardiomyocyte hypertrophy, suggesting its dual role in coordinating cell cycle exit and growth adaptation. Given our previous demonstration that CEND1 activates p53 signaling in cardiomyocytes 19, we propose a mechanistic model wherein CEND1-mediated p53 upregulation simultaneously represses cell cycle progression through canonical p21 activation, and promotes hypertrophic growth via induction of metabolic reprogramming 29, 30. This duality mirrors the established role of p53 in orchestrating both proliferative arrest and compensatory hypertrophy in pressure-overloaded hearts 31, 32, though further studies are needed to dissect CEND1-specific contributions to these processes. Together, our findings demonstrate that *Cend1* is a prominent, negative regulator of cardiomyocyte proliferation and that is suppressed by LSD1, although other factors may be at play. For example, *Mahmoud et al*. reported that the transcription factor MEIS1 negatively regulates cardiomyocyte cycling and that *Meis1* deletion could induce cardiomyocyte proliferation in mice after P7 9. When considered together, it is plausible that CEND1 and MEIS1 would act as brakes for cardiomyocyte cycling in the context of heart tissue regeneration and repair. In addition, multiple positive regulators of cardiomyocyte cycling such as YAP1 33, GATA4 34, Tbx20 35, 36, NRG1 37, IGF-1, HGF 38, Pkm2 39, PPAR 40, ERBB2 41, 42, FSTL1 43, mir302-367 44, mir-31a-5p 45, has-miR-590 46, has-miR-199a 47 have also been identified to be critical for heart repair through enhanced cardiomyocyte cycling. In this light, we speculate that the mechanism driving sufficient cardiomyocyte proliferation in adult mammalian hearts might be achieved through a gene therapy approach that simultaneously activates proliferation alongside de-repression of the negative regulators for cardiomyocyte cycling.

Heart repair involves many molecular and cellular processes including inflammatory response, immune cell migration and activation, cardiomyocyte proliferation as well as angiogenesis 48. In the present study, transcriptomic profiling of *Cend1*^-/-^ hearts revealed that the upregulated genes in *Cend1*^-/-^ mice were involved in G1/S phase transition, vasculature development and macrophage activation. Immunostaining experiments confirmed that there were more vessels and M2 macrophages in the hearts of *Cend1*^-/-^ mice, compared to controls. Enhanced angiogenesis is known to promote heart regeneration and repair following myocardial infraction in several animal models 49. In addition, M2 macrophages can promote heart regeneration and repair through their clearance functions as well as paracrine signaling 24, 50. Together with our current findings, we surmise that *Cend1* deletion led to promotion of heart repair through elevated cardiomyocyte proliferation, angiogenesis and macrophage activation. However, the precise molecular mechanisms underlying CEND1-mediated gene expression regulation remain to be elucidated and represent an important direction for future investigation. To further dissect the cell-autonomous functions of CEND1, subsequent studies employing cell type-specific conditional knockout models (targeting cardiomyocytes, endothelial cells, fibroblasts, and macrophages) will be particularly valuable for understanding its compartmentalized roles in cardiac regeneration.

The composition of extracellular matrix (ECM) is highly dynamic and associated with cardiomyocyte proliferation during heart development and repair following injuries 51, 52. Several ECM proteins can induce cardiomyocyte proliferation and promote heart regeneration following myocardial infraction in mammalian models. Agrn, mainly produced by endothelial cells, was the first identified ECM protein which promotes heart regeneration following myocardial infraction in mice 53 and pigs 22. More recently, Versican 23 and SPP1 24, respectively derived from fibroblasts and macrophages, have been shown to induce heart regeneration and improve cardiac function following myocardial infraction in mice. Interestingly, the expression levels for these three ECM proteins were significantly elevated in the hearts of *Cend1*^-/-^ mice. Future studies will define the mechanisms through which CEND1 regulates the expression of *Agrn*,* Versican* and *Spp1* to influence heart repair following injury.

Our study demonstrates that LSD1 overexpression or CEND1 deletion enhances cardiac regeneration in both neonatal and adult mouse models. While neonatal mice exhibited more robust regenerative capacity consistent with known developmental windows of cardiac plasticity, the observed functional improvements in adult mice - though statistically significant - warrant cautious interpretation due to fundamental differences between murine and human cardiac biology and the inherent limitations of adult mammalian regeneration. These findings provide important mechanistic insights into the LSD1/CEND1 regulatory axis in cardiac repair while highlighting the need for further validation in higher mammalian systems before considering clinical translation. These results nevertheless identify LSD1 activation/CEND1 inhibition as promising new directions for developing therapeutic strategies to promote endogenous cardiac repair in humans.

## Methods

### Animals

Mice subjected to neonatal cardiomyocyte-specific *Lsd1* knockout (*Lsd1*^MKO^) were generated by injecting AAV9-*cTnT*^cre^ (10 μl per pup) into the left ventricle of hearts of *Lsd1^f/f^* mice *54* at Postnatal day 1 (P1). Mice subjected to cardiomyocyte-specific *Lsd1* overexpression (*Lsd1*^OE^) were generated by injecting AAV9-*cTnTP*-*Lsd1* into the left ventricle (10 μl per pup) of hearts of P7 wild-type (WT) mice or through tail vein (100 μl per mice) of adult WT mice at 8 weeks. Mice subjected to cardiomyocyte-specific *Cend1* overexpression (*Cend1*^OE^) were generated by injecting AAV9-*cTnTP*-*Cend1* into the left ventricle (10 μl per pup) of hearts of WT mice at P1. The mice injected with equal volume of AAV9-GFP virus were used as controls. *Cend1*^-/-^ mice were purchased from GemPharmatech Co., Ltd and crossed with* Lsd1^f/f^* mice to generate *Lsd1^f/f^; Cend1*^-/-^ mice which were subjected *Lsd1* and* Cend1* double knockout (DKO) in heart tissues by injecting AAV9-*cTnT*^cre^ (10 μl per pup) into their left ventricle of hearts at P1. Mice care and experimentation procedures were carried out using protocols authorized by the Institutional Animal Care and Use Committee (IACUC) of Xian Jiaotong University. All mice were housed under typical environmental conditions of 45-65% relative humidity, temperatures of 21-24 °C, a 12 h light/dark cycle and free access to water and food in the animal Research Center of Xian Jiaotong University.

### Apical resection

Apical resection (AR) was performed on P1 or P7 mice as described previously 5. Briefly, neonatal pups were anesthetized by ice freezing for 5-10 min and then subjected to intercostal incisions between the third and fourth intercostal space to separate the pericardium and expose the heart. Injury was induced by surgically cutting the left ventricle apex (~15% of the ventricular myocardium). Finally, the chest wall and skin incision were both closed with 7-0 sutures. The neonatal pups of the sham group underwent the same procedure without apical resection. After surgery, all neonatal pups were transferred onto 37℃ heating pads for resuscitation.

### Histology and immunostaining

Mouse hearts were collected and fixed in 4% paraformaldehyde for 24-48 h at 4°C and then processed for paraffin embedding. The heart tissues were cut at a 5 μm thickness. Masson's trichrome staining and Hematoxylin and Eosin (H&E) were performed according to standard procedures. For immunostaining, tissue sections were prepared from paraffin-embedded tissue samples and dewaxed, then rehydrated through a series of graded ethanol washes with increasing proportional volumes of water, and followed by antigen retrieval through boiling with sodium citrate buffer (Sigma, S1804) (pH 6.0). The tissues were then blocked with 5% horse serum (Solaribo, SL042) at room temperature (RT) for 1 h and subsequently incubated with primary antibodies (Table S1) diluted in blocking buffer at 4 ℃ overnight. The following day, tissues sections on the glass slides were incubated with fluorescent-labelled secondary antibodies at RT for 1h. Nuclear counter staining was performed with DAPI staining (Beyotime, C1006). The stained slides were subjected to image acquisition using a confocal microscope.

### Western blotting

Heart tissues were homogenized and lysed in RIPA buffer containing protease inhibitors. After spinning at 4°C for 15 minutes, supernatants were collected and subjected to protein concentration quantification through a BCA assay kit. Defined quantities of lysates were denatured with SDS loading buffer**,** separated by 10% polyacrylamide gel electrophoresis and transferred to PVDF membranes. The membranes were then blocked in 5% BSA in Tris buffered saline with Tween-20 (TBST) solution for 1 h at RT and incubated with primary antibodies diluted in TBST containing 5% BSA at 4°C overnight. On the following day, the membranes were washed with TBST for three times and incubated with second antibodies conjugated with HRP at RT for 1h. After three times of washing with TBST, the membranes were subjected to color development by using an ECL detection kit and the chemiluminescence immune detection system.

### RNA extraction and quantitative PCR (qPCR)

Total RNA was isolated from mice hearts using the Trizol solution (Invitrogen 9088901). For cDNA synthesis, 1 μg of total RNA was reverse transcribed with a HiScript II reverse transcriptase Kit (Vazyme R222). Then qPCR was performed using the SYBR Green PCR Master Mix (Genstar A304-05) and gene-specific primers (Table S2). Results were calculated by using the 2-^△△^CT method and standardized by glyceraldehyde-3-phosphate dehydrogenase (GAPDH).

### RNA-seq data analysis

Heart tissues were collected from P14 control, *Lsd1*^MKO^ and *Cend1*^-/-^ mice and subjected to RNA-sequencing. The data were processed and analyzed as described previously 19. In brief, the raw sequencing reads were first assessed for quality using Fastp (version 0.23.1) 55 and selected for further analysis with the following excluding criteria: (1) containing adapter contamination, (2) more than 10% of bases are uncertain, (3) the proportion of low quality (Phred quality < 5) bases is over 50%. Next, the selected reads were mapped to the mouse reference genome (GRCm39) using the Hisat2 (v2.0.5) 56. The read numbers and FPKM for each gene were calculated using FeatureCounts (v1.5.0-p3) 57. After this, differential expression genes (DEGs) were identified using DEseq2 package47 in R 4.2.1, and p values were adjusted using the Benjamini and Hochberg's approach. Following exclusion of genes with less than 50 counts, genes of significantly differential expression were determined with padj ≤0.05 and |log2 (foldchange)| ≥ 0.5. Finally, The DEGs were subjected to Gene Ontology (GO) and KEGG pathways enrichment analysis using the clusterProfiler package (R 4.2.1). GO and KEGG terms with corrected *p* < 0.05 were considered significantly enrichment. Gene Set Enrichment Analysis (GSEA) of the complete pre-ranked gene list was carried out using the GSEA_4.1.0 software and the Hallmark gene set collection within the Molecular Signatures Database (MSigDB).

### Echocardiography

Mice were first anesthetized by inhalation of isoflurane mixed with pure oxygen at a concentration of 2% to 3% for induction of anesthesia and 1.5% to 2% for maintenance of anesthesia. Then, body hair from the left precordial chest region as removed using depilatory cream. Transthoracic echocardiography was performed using the VINNO 70 (VisualSonics) equipped with an X10 transducer operating at 23 MHz frequency. All measurements were obtained under light anesthesia (1.5% isoflurane) with maintained body temperature at 37°C. Standard parasternal long-axis and short-axis views were acquired at the papillary muscle level, with all analyses performed by an investigator blinded to experimental groups. The ejection fraction (EF) and fractional shortening (FS) features were both measured on M-mode images.

### Experimental Myocardial Infarction

Adult mice were subjected to surgical ligation of the left anterior descending coronary artery (LAD) to establish myocardial infarction (MI). In brief, the mice were anesthetized by inhalation of isoflurane mixed with pure oxygen. Subsequently, a 1 cm incision was made in the precardiac area of the mice, followed by blunt separation of the anterior thoracic muscle and intercostal muscles. Then gently rotating forceps were inserted into the chest through the left anterior 3-4 costal space. A 6-0 silk thread was ligated approximately 2 mm below the left atrium at a depth of 2-3 mm. The successful ligation was confirmed by observing the color change of distal vessel from red to pale. Finally, the chest and skin incision were closed by using 4-0 silk sutures. The mice were warmed up on a heating pad at 37°C for several minutes until recovery.

### Statistical analysis

All statistical analyses were conducted using Graph Pad Prism 9.0 software. All experimental results were from at least three biological replicates and presented as mean ± standard deviation. Comparison between two groups were conducted using a two-tailed Student's *t*-test, while one-way analysis of variance was used for comparisons among multiple groups. A *p-*value less than 0.05 was considered statistically significant.

## Supplementary Material

Supplementary figures and tables.
