# Supplementary material for: Regulation of heart regeneration by LSD1 through suppressing CEND1
Source: Theranostics. 2025 May 25;15(13):6313–28. doi: 10.7150/thno.110297 (PMC12159839; doi:10.7150/thno.110297)
Supplement: Supplementary file 1 — Supplementary figures and tables. [file thnov15p6313s1.pdf]

Supplemental Figures and Tables

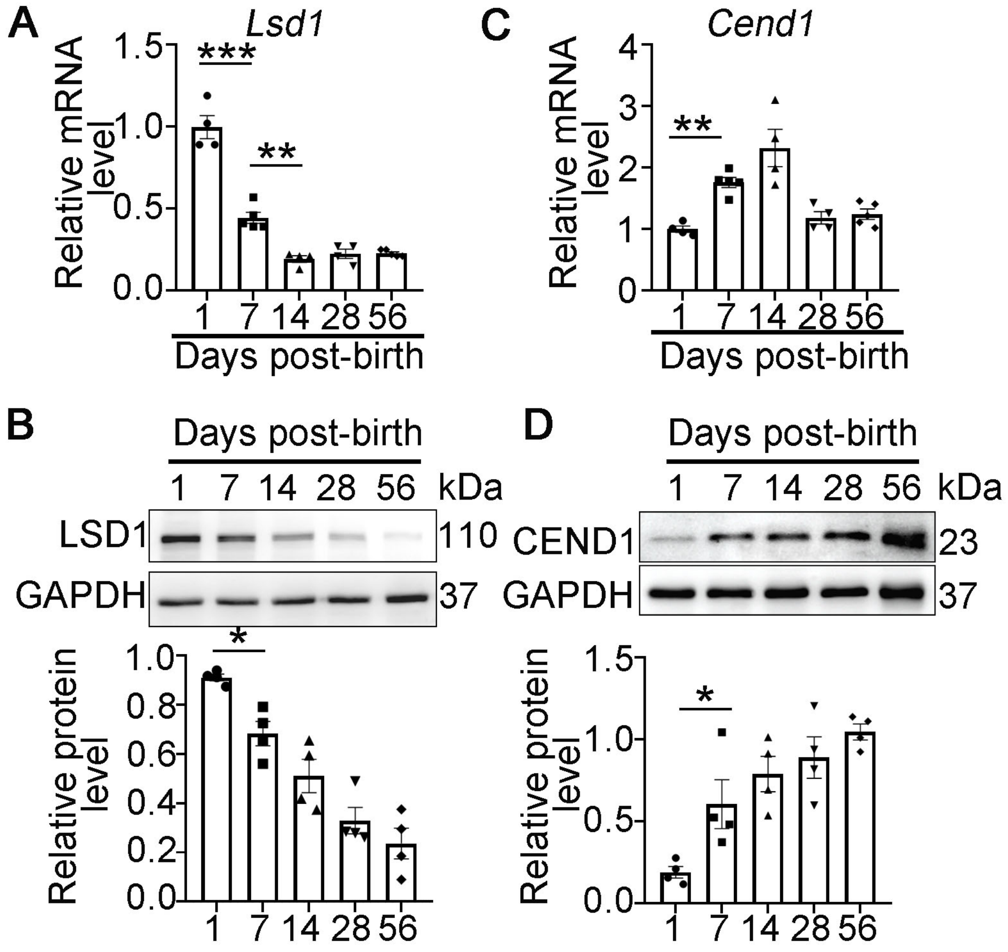

Figure S1. Complimentary expression of LSD1 and CEND1 in postnatal murine heart tissues. (**A, C**) qPCR analysis of *Lsd1* (**A**) and *Cend1* (**C**) mRNA levels in hearts of mice at indicated stages (n = 4-5/group). (**B, D**) Western blotting analysis of LSD1 (**B**) and CEND1 (**D**) protein levels in hearts of mice at indicated stages (n = 4/group).

*p < 0.05, **p < 0.01, ***p < 0.001 by unpaired student′ s *t*-test.

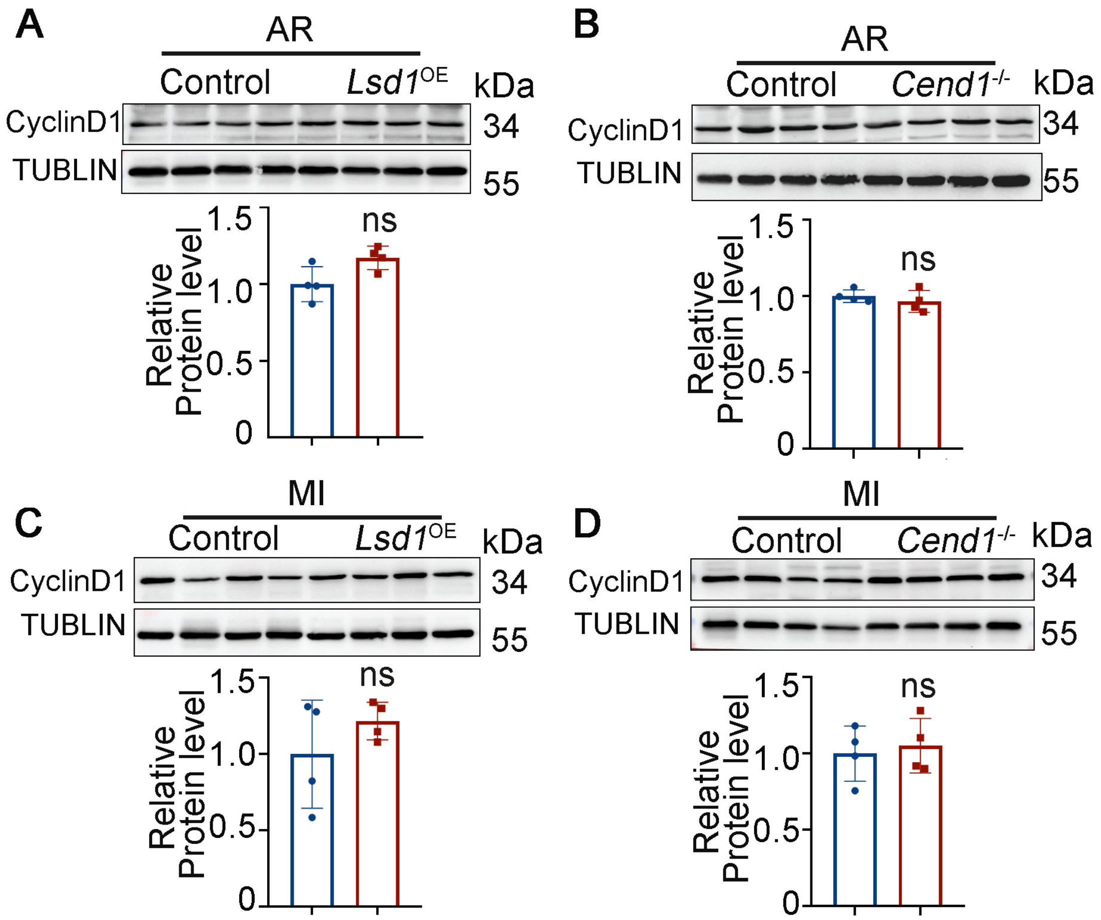

Figure S2. **Cyclin D1 protein levels were unaffected by genetic manipulations of *Lsd1* or *Cend1* following heart injuries.** (**A-D**) Western blotting analysis of CyclinD1 protein levels in the hearts of *Lsd1*^{OE} (**A, C**) and *Cend1*$^{-/-}$ (**B, D**) mice following apical resection (AR) at neonatal stage (**A, B**) and myocardial infraction (MI) at adult stage (**C, D**). (n = 4/group). ns, no significance by unpaired student's *t*-test.

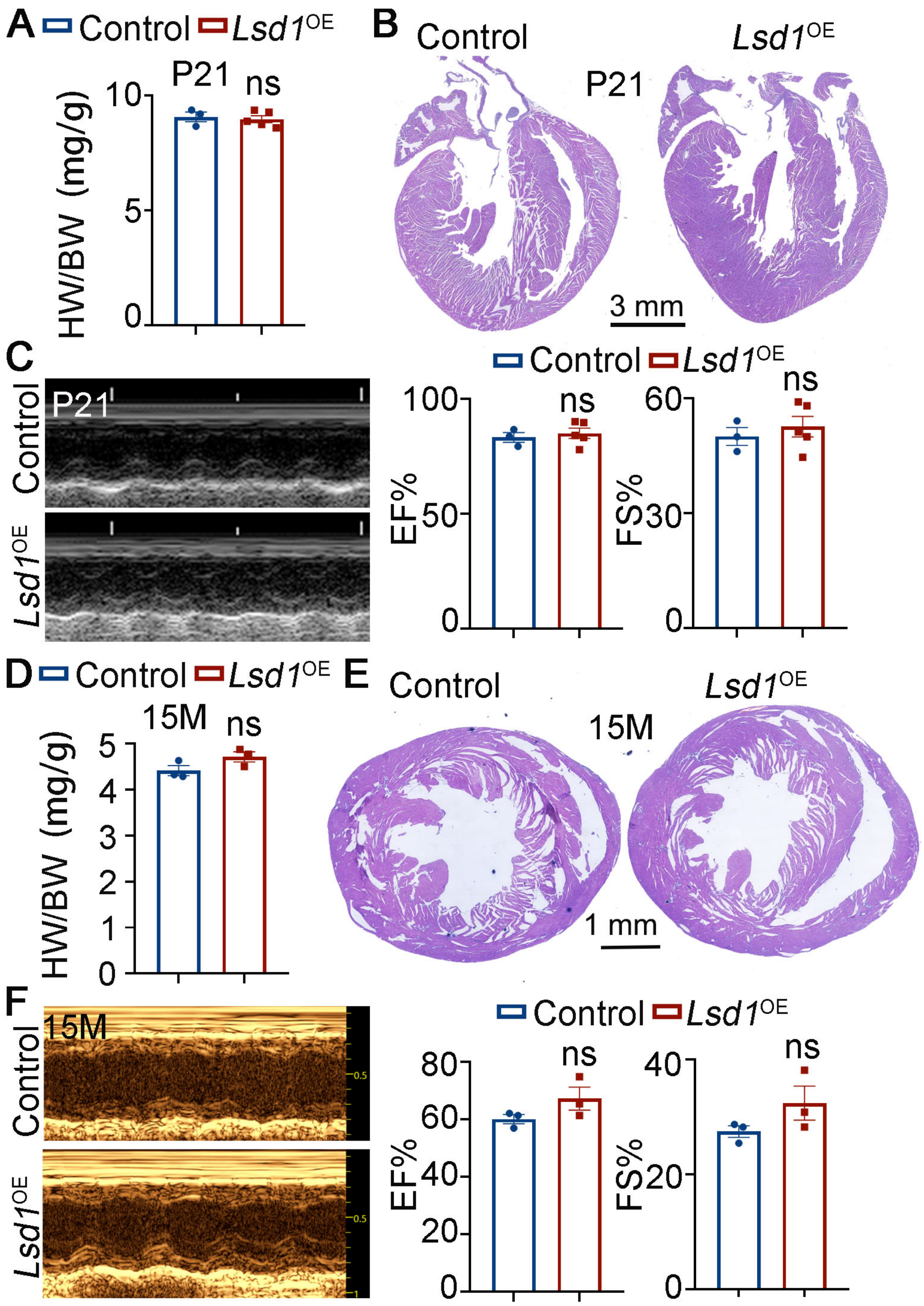

Figure S3. LSD1 overexpression does not alter cardiac structure or function in mice. (A) Heart weight-to-body weight (HW/BW) ratios in P21 *Lsd1*^{OE} (n = 5) and control (n = 3) mice. **(B)** Representative hematoxylin and eosin (H&E)-stained heart sections from P21 mice. **(C)** Representative echocardiograms from P21 mice and quantification of ejection fraction (EF) and fractional shortening (FS) in control (n = 3) and *Lsd1*^{OE} (n = 5) groups. **(D)** HW/BW ratios in 15-month-old (15 M) mice (n = 3/group). **(E)** H&E-stained heart sections from 15 M mice. **(F)** Representative echocardiograms from 15 M mice and quantification of EF and FS (n = 3/group). ns, no significance by unpaired student's *t*-test.

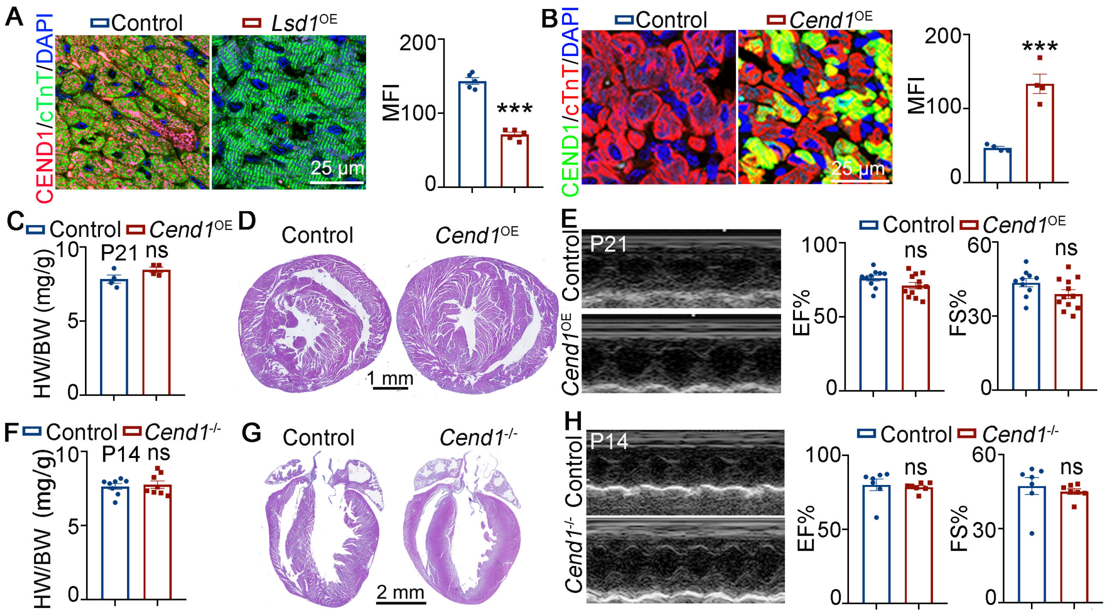

Figure S4. Cardiac structure and function are unaffected by *Cend1* genetic manipulation. (A) Co-immunostaining of CEND1 and cTNT antibodies in P14 *Lsd1*^{OE} and Control hearts (n = 5/group). The mean fluorescence intensity (MFI) was quantified and shown. **(B)** Co-immunostaining of CEND1 and cTNT antibodies in P14 *Cend1*^{OE} and Control hearts (n = 4/group). MFI was quantified and shown. **(C)** Heart weight-to-body weight (HW/BW) ratios in P21 mice (n = 4/group). **(D)** Representative H&E-stained heart sections from P21 mice. **(E)** Representative echocardiograms from P21 mice and quantifications of ejection fraction (EF) and fractional shortening (FS) in Control (n = 10) and *Cend1*^{OE} (n = 12) groups. **(F)** HW/BW ratios in P14 *Cend1*$^{-/-}$ and Control mice (n = 8/group). **(G)** H&E-stained heart sections from P14 *Cend1*$^{-/-}$ and Control mice. **(H)** Representative echocardiograms from P14 mice and quantifications of EF and FS in *Cend1*$^{-/-}$ (n = 7) and Control (n = 7) groups. ns, no significance, ***p < 0.001 by unpaired Student's t-test.

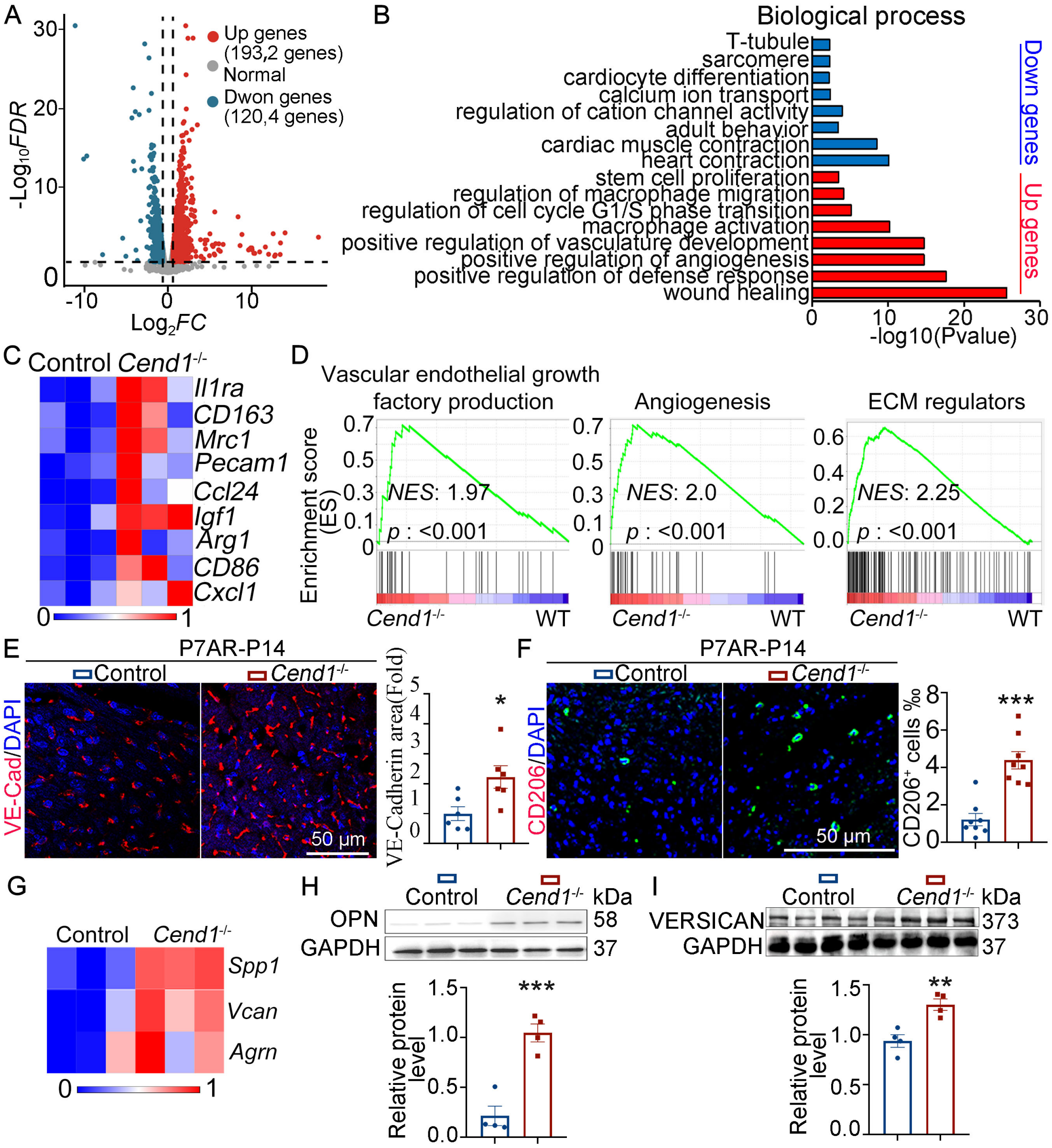

Figure S5. Loss of *Cend1* leads to gene expression alterations associated with cell proliferation, angiogenesis, and macrophage polarization. (**A-D**) Heart ventricle tissues were isolated from P14 control and *Cend1*$^{-/-}$ mice which received apical resection (AR) surgeries at P7 and subjected to RNA-sequencing analysis. Volcano plots showing the differentially expressed genes (**A**). The chart showing the encriched biological processes revealed by Gene ontology (GO) analysis (**B**). A Heatmap showing the expression of M2 macrophage markers (**C**). The charts showing the enriched biological processes revealed by Gene Set Enrichment Analysis (GSEA) (**D**). (**E**) VE-cadherin immunostaining marking vascular endothelial cells within apex regions (n = 6/group). (**F**) Immunostaining for CD206 indicating M2 macrophages (n = 8/group). (**G**) Heatmap showing the expression of ECM genes (*Spp1*, *Vcan* and *Agrin*) known to be involved in heart regeneration. (**H, I**) Western blotting analysis of OPN (encoded by *Spp1*) and Versican (encoded by *Vcan*) protein levels in P14 hearts (n = 4/group). **p < 0.01, ***p < 0.001 by unpaired student's t-test.

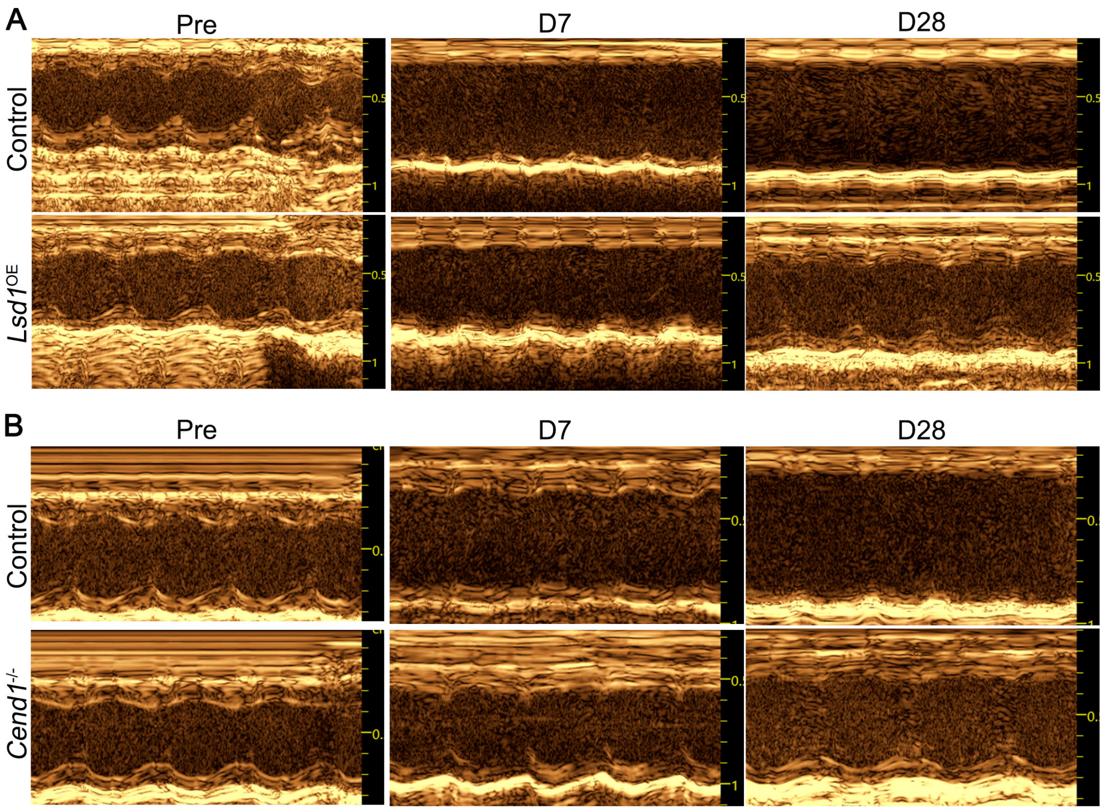

Figure S6. Echocardiographic assessment of cardiac function in *Lsd1*^{OE} and *Cend1*$^{-/-}$ mice pre- and post-myocardial infarction (MI). Representative echocardiographic images of ***Lsd1***^{OE} **(A)** and *Cend1*$^{-/-}$ **(B)** mice at indicated time points. Pre, pre-MI; D7, Day 7 post-MI; D28, Day 28 post-MI.

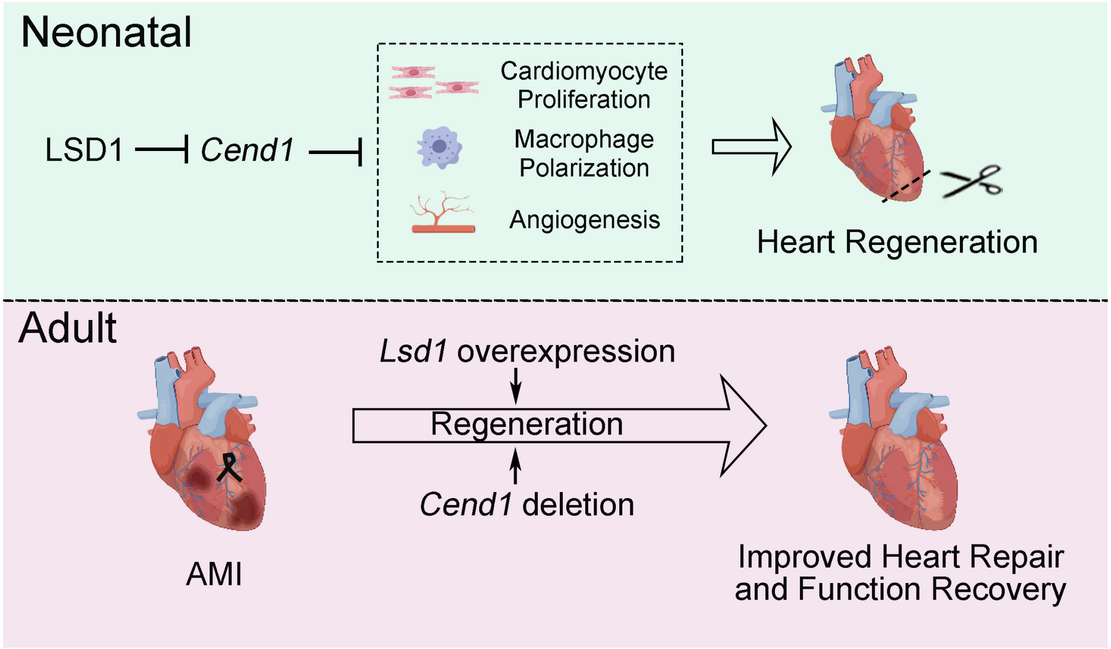

Figure S7. Diagrammatic summary for an LSD1-CEND1 signaling axis in neonatal and adult heart regeneration and repair. LSD1-dependent suppression of *Cend1* is essential for heart regeneration in neonatal mice. Mechanistically, *Cend1* negatively regulates cardiomyocyte proliferation, macrophage polarization and angiogenesis. Notably, both *Lsd1* overexpression and *Cend1* deletion can promote cardiac repair and functional recovery following myocardial infraction in adult mice, indicating that proper balanced achieved through regulation of a LSD1-CEND1 signaling axis may serve as therapeutic approach for heart disease in mammals.

Table S1. A list of antibodies used in this study.

Antibody	Company	Cat#	Source	WB dilution	IF dilution
AuroraB	abcam	ab2254	Rabbit	N/A	1:300
CEND1	CST	8944S	Rabbit	1:1000	1:200
cTnT	Proteintech	15513-1-AP	Rabbit	N/A	1:500
cTnT	Abcam	Ab8295	mouse	N/A	1:500
CyclinD1	Proteintech	60186-1-Ig	Mouse	1:3000	N/A
GAPDH	Proteintech	10494-1-AP	Rabbit	1:5000	N/A
Ki67	Abcam	ab15580	Rabbit	N/A	1:400
LSD1	CST	2139S	Rabbit	1:1000	1:200
OPN	CST	88742S	Rabbit	1:1000	N/A
PCNA	Abcam	Ab29	Mouse	1:1000	N/A
pH3	CST	9701S	Rabbit	N/A	1:400
VE-cadherin	R&D	AF1002	Goat	N/A	1:400
Versican	ABclonal	A19655	Rabbit	1:1000	N/A
α-Tublin	CST	2125S	Rabbit	1:1000	N/A

Table S2. A list of primers used in this study.

Mus gene	Forward sequence	Reverse sequence
Lsd1	CCAGGGATCGAGTAGGTGGA	GGAACAGCTTGTCCATTGGC
Cend1	GAAGACACCAGCCAAGGCAGAT	CTCCAGTGTTGGACTCGTCCTC
